# 1876. COVID-19 Reinfection and Disease Severity in the New York City Health + Hospitals System

**DOI:** 10.1093/ofid/ofac492.1503

**Published:** 2022-12-15

**Authors:** Jenny R Smolen, Thomas D Filardo, Annie George, Sakil Bhuiyan, Sowmya Kalava, Noor Shahin, Jonathan Farkas, Jazila Mantis, Merjona Saliaj, Vikramjit Mukherjee, Carlos Salama, Benjamin Eckhardt, Gabriel Cohen

**Affiliations:** NYC Health + Hospitals, New York City, New York; New York City Health+Hospitals / Bellevue, New York, New York; New York City Health+Hospitals, New York City, New York; NYCHHC Queens Hospital Center, Brooklyn, New York; Elmhurst Hospital-Icahn School of Medicine, Elmhurst, New York; Internal Medicine at Mount Sinai - Elmrhust Hospital Center, Haledon, New Jersey; New York University / Bellevue Hospital Center, NewY, New York; Mount Sinai, Jamaica, New York; Queens Hospital Center/Mount Sinai, Jamaica, New York; nyusom, NY, New York; Elmhurst Hospital / Icahn School of Medicine, Elmhurst, New York; NYU School of Medicine, New York, New York; NYC Health + Hospitals, New York City, New York

## Abstract

**Background:**

Though reinfection with SARS-CoV-2 is well documented, there remains uncertainty about the potential for more severe symptoms with reinfections compared to index infections.

**Methods:**

Patients who received SARS-CoV-2 PCR testing between March 1, 2020 and March 1, 2021 at New York City Health and Hospitals (NYC H+H) facilities and had two positive tests > =90 days apart were included in the analysis. Clinical and demographic data were extracted from the electronic medical record. Manual chart review was done to confirm symptomatology, assess COVID-19 related hospital admissions, and determine WHO disease severity. Patients were then classified as unlikely reinfection, possible reinfection, or probable reinfection based on symptomatology, PCR and antibody testing, and lack of alternative diagnoses. Patients were classified as “unable to be assessed” if symptomatology could not be assessed for both episodes of PCR positivity.

**Figure 1:**
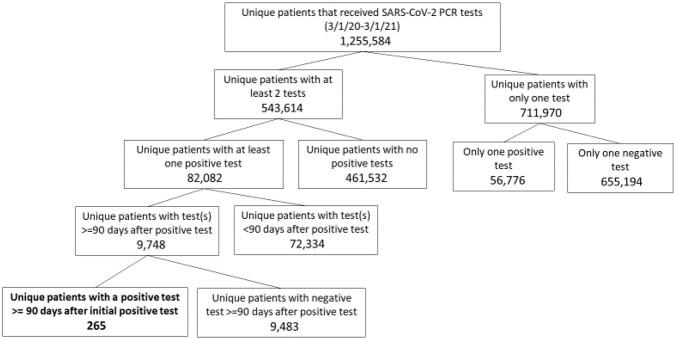
Flow chart of selection process for study inclusion

**Figure 2:**
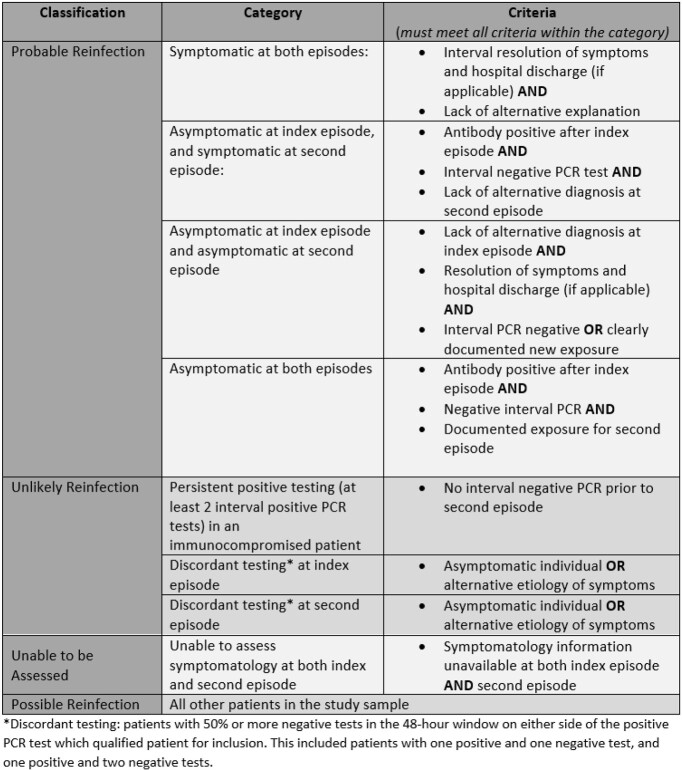
Reinfection Classification

**Results:**

During our study timeframe, 1,255,584 unique patients received at least one SARS-CoV-2 PCR test, 265 of whom had two positive tests > =90 days apart. We categorized 20 patients as unable to be assessed, 28 as unlikely reinfection (1 persistent PCR positivity, 27 unlikely true infection at index or second PCR-positive episode), and 217 as possible or probable reinfection. Of the 217, at their index episode 79 had an asymptomatic infection (36.4%) and 17 were severe or critical (7.8%). At their second episode, 162 patients had an asymptomatic infection (74.7%), and 5 were severe or critical (2.3%).

Only 24 patients with possible/probable reinfection had a more severe COVID reinfection than index infection, and 20 of the 24 had asymptomatic index infections. Three patients were hospitalized at both episodes, and two deaths possibly attributable to COVID-19 reinfection were noted in this cohort.

**Figure 3:**
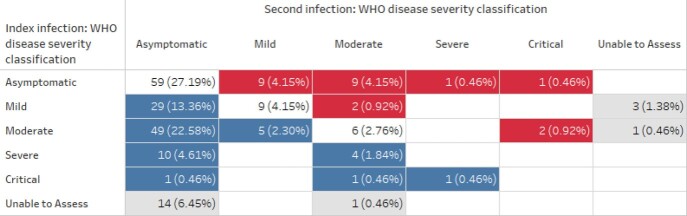
Change in WHO disease severity classification from index to second infection among probable/possible reinfection cases (n=217)

Red indicates increase in disease severity from index to reinfection (n=24), blue indicates decrease in disease severity from index to reinfection (n=100), white indicates no change (n=74) and gray indicates unable to assess disease severity at index or second infection (n=19).

**Conclusion:**

COVID-19 reinfection was rare in a high incidence setting among patients tested at NYC H+H facilities. Disease severity was generally milder in reinfection, although severe and critical disease occurred in a small number of patients. These findings from earlier in the pandemic (presumably wild-type and alpha variant) provide data for comparison in understanding how reinfection is evolving with newer variants.

**Disclosures:**

**Carlos Salama, MD**, Genentech: Advisor/Consultant **Gabriel Cohen, MD**, Daybreak Health: Advisor/Consultant|Daybreak Health: Board Member|Daybreak Health: Ownership Interest.

